# Mr. Cooper on the Sight

**Published:** 1847-04

**Authors:** 


### Art. XV.

*Practical Remarks on Near Sight, Aged Sight, and Impaired Vision; with Observations upon the Use of Glasses and on Artificial Light.* By William White Cooper.—*London*, 1847. 8vo, pp. 216.

We cannot agree with Mr. Cooper, that there is a "paucity of works of authority in the English language, upon Near and Aged Sight, the use of Glasses, and Impaired Vision." Beer's Pflege have long been familiar to the English reader, under the spurious title of 'The Art of Preserving the Sight, by an Experienced Oculist.' Adams's 'Essay on Vision,' Kitchener's 'Economy of the Eyes,' Ross 'on the Use and Abuse of Spectacles,' Walker's 'Philosophy of the Eye,' Franz 'on the Eye,' Hunter 'on the Influence of Artificial Light,' and many other works might be mentioned, which expressly embrace the subjects which Mr. Cooper proposes "to elucidate." We should still be glad, however, to hail a new and more complete work on the preservation of sight, than any that has hitherto appeared; and that whether it was intended for the popular reader's instruction, or for that of the medical practitioner.

Mr. Cooper's work is divided into six chapters, which treat of Light, Myopia, Presbyopia, Spectacles, Inflammation and other diseases of the Eye and their treatment, and Artificial Light. Upon these various topics there is much information put down in Mr. Cooper's pages; but as he has evidently had in his eye, as worthy of imitation, our old familiar friend, who aimed to kill two dogs with one stone, what he says is half addressed to the popular reader, half to the medical, and we fear will be found satisfactory to neither. For example, he gives in his first chapter, an account of the anatomy of the organ of vision. Now, neither of the above parties will thank him for so scanty a statement respecting the nerves of the iris, or the lenticular ganglion as that contained (page 29) in this single short sentence—"The nerves which supply the iris are derived from the lenticular ganglion." "So we have often been told," will the medical reader be disposed to remark, "and we know very well how the lenticular ganglion is made up of a sensitive root and a motive root, and how its branches, the ciliary nerves, sink into the annulus albidus of the choroid; but since Mr. Cooper was in an "elucidating" mood when he wrote his book, we wish he had thrown some light on the final distribution of these nerves, which, if they go at all to the iris, send but a very small proportion of their filaments to that membrane." "Lenticular ganglion!" exclaims the non-professional reader. "What is that? The nerves of the iris are derived from it? I am just as wise as when I began." This may serve to show the futility of works on anatomy recommended for popular use, but written in the sketchy manner of Mr. Cooper.

We are not satisfied that the scientific parts of Mr. Cooper's work will be found everywhere correct, nor that their faults will escape detection even by the merest tyro in medicine. His frontispiece, for instance, is, he says, a vertical section of the eye, but this must be a mistake, as the optic nerve is represented entering the eye much below the axis. At page 15, he tells us, that the crystalline lens is "the most important of the refracting media of the eye;" whereas every well-informed medical student knows, that the crystalline does not refract the light entering the eye nearly so much as the cornea, and that, although the crystalline may be removed, and yet the person continue to see, we cannot take the same liberty with the cornea or the vitreous humour, without destroying vision. Even evacuation of the aqueous humour will, till that fluid is regenerated, affect sight much more than removal of the crystalline. At page 35, Mr. Cooper says, "If, before a hole made in the window-shutter of a darkened room, a double convex lens be placed, an inverted picture of the scene without will be portrayed by the lens upon a piece of white paper." Now, the merest tyro knows that the inverted picture is portrayed before the lens is applied to the hole; in other words, that the hole forms the picture, which the lens merely renders brighter and smaller. At page 37, he tells us, that "for *single vision* it is essential that our eyes be so adjusted, that the image formed in each shall fall on corresponding points of the retina." Physiologists have given the name of *corresponding points* to those parts of the two retinæ which lie at equal distances from the vertex, and in the same direction ; that is, both to the right, or both to the left, both upwards, or both downwards. Had Mr. Cooper studied Professor Wheatstone, to whom he immediately afterwards refers, he would have learned that the observations of that gentleman afford ample proof, that objects, the images of which do not fall on corresponding points of the two retinæ, may still appear single. If Mr. Cooper will just hold out a pencil-case, or any similar body, directly forwards from the point of his nose, he will see it single, by means of two images, falling the one on the temporal side of the right retina and the other on the temporal side of the left, and therefore on non-corresponding points. What is still more curious, Professor Wheatstone has shown, that similar images falling on corresponding points of the two retinæ may appear double and in different places ; all which is familiar to every one who has amused himself with the professor's instrument called the *stereoscope*.

CHAPTER V is a strange medley, consisting of cases of muscæ volitantes, and amaurosis ; Milton's Letter to Leonard Phalaras the Athenian, (who, residing at Paris as envoy from the Duke of Parma, had got acquainted with Thevenot, the oculist), and receipts for curing ophthalmia tarsi. These last can be of no use, as here set down, to medical men, and may be the source of much evil to patients should they take it in their heads to *try* to cure themselves by means of the six formulæ, here clapt so unceremoniously cheek-by-jole with the immortal poet's learned epistle.

At page 51, we have a case narrated of incomplete congenital cataract, for many years mistaken for aggravated myopia. Mr. Cooper dilated the pupil by atropine, and the patient saw past the cataractous portion of the lenses. A very trifling operation, he adds, rendered the newly acquired powers of vision permanent. Mr. Cooper should have stated what the operation was, which, whether it was prolapsing a bit of iris through the cornea or dividing the cataract, he might have done almost in as few words as he has used to raise the reader's wonder by calling it "a very trifling operation."

We have no great opinion of the application of collyria to the surface of the conjunctiva. By removing the mucous secretion of that membrane, they generally irritate the eye rather than relieve it, except where the secretion is morbidly changed in quantity and quality. Mr. Cooper thinks differently, and therefore advises the use of an eye-cup, adding that if one "cannot be procured, the eye should be bathed with soft rag, dipped in the collyrium." We should prefer the rag, but an egg-cup will form no bad substitute for an eye-cup, if such a contrivance is thought essential.

At page 157, the reader will find a sufficient specimen of what we deem the most objectionable parts of Mr. Cooper's book, in the directions laid down for the treatment of congestion of the retina. These embrace nothing but what is perfectly familiar to the practitioner, but they would be extremely dangerous for any patient to attempt to follow, without medical superintendence. Even the dietetical advice is not at all to our mind, for assuredly dyspeptic subjects, troubled with flatulence and acidity, will be far better without such stimulants as India pale ale, porter or stout, while weak mixtures of spirit and water, such as soda or seltzer water, with a little brandy or genuine sherry, recommended by Mr. Cooper, from running so readily into acid, are the very things to be especially avoided.

On the whole, notwithstanding these objections, we have no hesitation in saying, that Mr. Cooper's work contains many just and valuable hints on the preservation of sight. The instructions he delivers on the necessity of caution in the use of glasses are highly important, and the book is written in a pleasant readable style.

---

